# Editorial: The Skin in Cancer Predisposition Syndromes

**DOI:** 10.3389/fmed.2021.774704

**Published:** 2021-11-08

**Authors:** Licia Turolla, Isabella Mammi

**Affiliations:** ^1^Medical Genetics Unit, Treviso Hospital, Treviso, Italy; ^2^Medical Genetics Unit, Dolo Hospital, Dolo, Italy

**Keywords:** skin, genetics, cancer, cancer predisposition syndrome, genodermatosis

About 10% of cancers occur in people who have mutations in one of the many cancer predisposition genes.

The suspicion of a “hereditary tumor” is generally placed on the basis of the patient's personal history (young age of onset, multiple synchronous, or metachronous tumors) or family history (two or more subjects with similar tumors in two or more generations).

Many hereditary tumors occur in subjects who also present non-neoplastic manifestations (cancer predisposition genetic syndromes). In many cases, these syndromes can be the result of “*de novo*” mutations and, therefore, a family history of tumors may be missing. The ability to recognize these conditions on the basis of non-neoplastic manifestations can allow early surveillance, with possible improvement of the prognosis, as well as the assessment of the risk of recurrence in the offspring.

The possibility of correlating a specific skin phenotype with the suspicion of a cancer predisposition genetic syndrome before cancer develops can motivate and guide the execution of a genetic test for diagnostic confirmation. The inclusion of genetic information in the patient's clinical management will allow the implementation of personalized surveillance and treatment measures, both for neoplastic and non-neoplastic manifestations that may be foreseeable.

This is well-reflected by the articles in the Research Topic “The Skin in Cancer Predisposition Syndromes”.

Zhao et al. in the Original Research article “Update of Clown Nose-Like Lesion, a Underrecognized Manifestation of Metastatic Malignancies and Genetic Cancer Predisposition Syndromes” highlight how the clown nose-like lesions, characterized by a reddish or skin-colored bulge of the tip of the nose or a bulbous tip of the nose, can serve as indicators to at least three categories of clinical issues: metastatic visceral tumors, genetic syndromes, and primary diseases involving the nasal tip.

The authors, on the basis of the cases obtained from the literature and their personal observations, describe the different behavior of the lesion in the various categories of pathologies. In particular, it appears that clown nose-like lesions caused by metastatic malignancies were often solitary and more common in male older individuals, appeared for a short time and were prone to be misdiagnosed as primary nasal diseases, leading to poor prognosis. Clown nose-like lesions associated with genetic cancer predisposition syndromes usually develop at a young age with female preference, and are accompanied by multiple systemic involvements; family history is often positive. These two kinds of clown nose-like lesions are often asymptomatic, which delays the diagnosis and treatment of underlying condition.

Some syndromes with a predisposition to cancer are particularly rare conditions and, therefore, poor information is available about them. This is the case with Clericuzio syndrome or Poikiloderma with Neutropenia. In their Case Report “Poikiloderma with Neutropenia and Mastocytosis: A Case Report and a Review of Dermatological Signs,” Piccolo et al. describe a patient with this rare condition, confirmed by the finding of homozygosity for the known variant c.243G>A (p.Trp81Ter) of the USB1 gene. The authors present a comparison table of the clinical data observed in their patient and in the other subjects reported in the literature with the same variant. In addition, they present a review of the literature with a focus on dermatological signs. The presence of pictures of the dermatological lesions, and a detailed description of the clinical course of the disease provide useful information that allow clinical diagnosis. Additional findings present in the patient described by Piccolo et al. (mastocytosis and speech delay) may be coincidental.

In their Mini Review “The Skin in Cowden Syndrome,” Lim and Ngeow provide a very comprehensive review of dermatological signs of Cowden syndrome. Each skin change is described and differential diagnosis for genetic causes is given. Two useful tables summarize differential diagnoses for the different skin manifestations. Criteria for considering genetic test for such condition is provided, as well as recommendations for surveillance. Moreover, therapeutic interventions and new potential targeted therapies for skin manifestations are also discussed.

In their Original Research article “PTEN Hamartoma Tumor Syndrome: Skin Manifestations and Insights Into Their Molecular Pathogenesis,” Innella et al. characterized a single-center series of 20 Italian patients and performed molecular assessment to explore the mechanisms involved in the pathogenesis of PTEN-associated manifestations, with special focus on mucocutaneous features. The clinical manifestations that led to the diagnostic suspicion were taken into consideration and the indication for genetic testing was discussed. The frequency of each clinical sign was assessed, with particular regard to mucocutaneous manifestations and oncological complications, and the types of variants observed in the PTEN gene were described. The molecular characterization of a trichilemmoma in which the wild-type PTEN allele was retained and expressed, allowed to reinforce the evidence that PTEN does not require a second somatic hit to initiate pathogenic processes. The observation of diverse cancer genes mutations in different tumor types lead the authors to hypothesize that the presence of a constitutional PTEN mutation and the consequent reduction of the PTEN dosage creates a “permissive” environment to the carcinogenic effect of somatic mutations in the cells.

The aim of this Research Topic was to increase the degree of knowledge and, consequently, of attention, toward the non-neoplastic skin manifestations that are part of the various cancer predisposition genetic syndromes. The articles of our collection provide information that allow the clinician to be able to recognize them as an element of suspicion of these conditions.

## Author Contributions

All authors listed have made a substantial, direct and intellectual contribution to the work, and approved it for publication.

## Conflict of Interest

The authors declare that the research was conducted in the absence of any commercial or financial relationships that could be construed as a potential conflict of interest.

## Publisher's Note

All claims expressed in this article are solely those of the authors and do not necessarily represent those of their affiliated organizations, or those of the publisher, the editors and the reviewers. Any product that may be evaluated in this article, or claim that may be made by its manufacturer, is not guaranteed or endorsed by the publisher.

